# A Study of Unusual Pacemaker Infection by Mycobacterium Tuberculosis in Indian Patients

**DOI:** 10.1016/s0972-6292(16)30817-8

**Published:** 2014-12-15

**Authors:** Amresh Kumar, Tanu Agrawal

**Affiliations:** 1 Department of Medicine (Cardiology unit), Sri Ram Murti Smarak Institute of Medical Sciences, Bareilly, U.P., India; 2 Department of Pathology, Sri Ram Murti Smarak Institute of Medical Sciences Bareilly, U.P., India

**Keywords:** Mycobacterium tuberculosis, Pacemaker Infection

## Abstract

**Background:**

The expanding clinical indications of cardiac rhythm management have led to an increased use of pacemaker implantation which is associated with increased incidence of pacemaker infections. *Staphylococcus aureus* and *epidermidis* account for the vast majority of pacemaker infections. Pacemaker infection due to Mycobacterium tuberculosis (*M. tuberculosis*) is very rare, only few cases having been reported till date.

**Methods:**

We describe here a study of three patients of pacemaker pocket infection with *M. tuberculosis*.

**Conclusion:**

The possibility of mycobacterial pacemaker infection should always be kept in mind in patients with delayed pacemaker infection.

## Introduction

Pacemaker infections with *M. tuberculosis* are extremely rare. The reactivation of latent tuberculosis (TB) at sites affected by trauma or surgery has been described [1-3]. Mycobacterial infections may present many years after a procedure, often in a non-specific fashion and without any visible sign of infection. Doherty et al [4] described a case of a pacemaker pulse-generator pocket infection in a patient with military tuberculosis while Hellwig et al [5] reported a case of pacemaker infection with *M. tuberculosis* in an 8 year old patient. Another reported case of tubercular infection involving epicardial pacing wires suggests that placement of epicardial wires may be associated with reactivation of previously silent pericardial infection [6].

In our institute from January 2007 to December 2013 seven hundred and thirty two pacemakers were implanted. We describe here three cases of delayed pacemaker infection (after one year) with *M. tuberculosis* in the absence of active pulmonary or disseminated disease, and review the literature of pacemaker infection by *Mycobacterium* species.

## Case 1

A 48-year-old man underwent implantation of a dual chamber pacemaker for complete heart block. The patient was a heavy smoker and chronic alcoholic. After a symptom-free interval of 15 months, the patient was seen with a subcutaneous abscess of the pacemaker site that was not associated with any systemic symptom or sign of infection. He did not report any other symptoms. On physical examination a small soft tissue swelling was noted at the incision line of the pacemaker site. The swelling was erythematous, non tender and there was no wound dehiscence or pus discharge. Blood cell counts were normal and blood cultures remained sterile. He was treated empirically with intravenous ampicillin and amikacin. After five days of therapy the swelling did not subside and spontaneous pus discharge started. Pus was negative by gram stain, and bacterial culture was sterile but it was positive for acid fast bacilli (AFB). Diagnosis of *M. tuberculosis* was confirmed by polymerase chain reaction (PCR) assay. There was no radiographic evidence of pulmonary TB. A transthoracic echocardiogram showed no evidence of pericardial disease or any vegetation. He was initially treated with isoniazid (INH) 300 mg, rifampicin 450 mg, ethambutol 800 mg and pyrizinamide 1500 mg daily for 3 months followed by INH 300 mg and rifampicin 450 mg daily for another 9 months. Healing of the wound occurred within 14 days of starting therapy. He responded well to treatment. After a follow up of 3 years the patient is asymptomatic.

## Case 2

**Figure 1:**
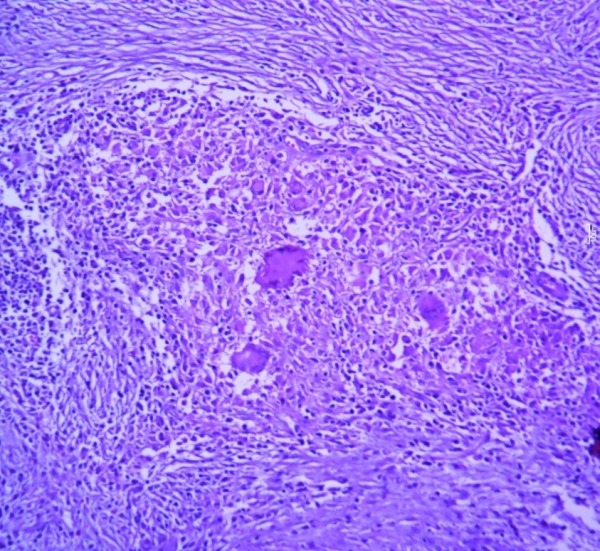
Microphotograph of H&E stained section of case 2 showing granuloma composed of epithelioid cells and Langhans giant cells.

A 70-year-old man presented with a subcutaneous abscess at the pacemaker site with pus discharge after 18 months of single chamber pacemaker implantation. Patient was a known diabetic for 20 years on oral anti-diabetic therapy with well controlled diabetes, and a heavy smoker. On physical examination a small soft tissue swelling was noted over the pacemaker site with pus discharge. Haematology and biochemical investigations were normal. After two blood culture samples were sterile, empirical intravenous amoxycillin and gentamycin were started and wound dressing was done with topical antibiotics. Pus was also sent for AFB staining which was negative, but PCR for *M. tuberculosis* was positive. Pacemaker pocket debridement and resuturing of wound was done. Tissue removed from the pocket during debridement was sent for histopathology which revealed granulomatous inflammation (Figure 1). The chest radiograph and echocardiography did not reveal any abnormality. He was initially treated for 3 months with isoniazid, rifampicin, ethambutol and pyrizinamide followed by isoniazid and rifampicin for another 9 months. After a follow up period of 3 years the patient is asymptomatic.

## Case 3

A 71-year-old, non diabetic female underwent implantation of biventricular pacemaker for complete heart block and left ventricular dysfunction. The patient had been successfully treated for pulmonary tuberculosis 15 years back. After a symptom-free interval of 60 months after device implantation, the patient presented with a large lump over the pacemaker site. On physical examination a 10 x 10 cm soft tissue swelling was noted over the pacemaker site. The swelling was non tender, non erythematous and there was no pus discharge. Blood cultures were sterile. Blood cell counts were normal. Ultrasound revealed a large pus collection in the pacemaker pocket. Pus was drained and sent for microbiological analysis. Pus was negative by gram stain, and aerobic and anaerobic cultures were sterile. Pus was also negative for AFB but PCR for *M. tuberculosis* was positive. There was no evidence of active pulmonary or extrapulmonary tuberculosis and three sputum samples were negative for AFB. The patient was started on quadruple treatment with rifampicin, isoniazid, ethambutol and pyrazinamide. Wound healing did not occur probably due to large size of swelling so the pacemaker device was explanted and patient was put on temporary pace maker. A new permanent pacemaker device was reimplanted at another site after 4 weeks of starting of ATT. The patient is now asymptomatic and presently continuing on ATT.

Table 1 summarizes the characteristics of the three cases.

## Discussion

Very few case reports of *M. tuberculosis* pacing system infections exist in the literature. The first is an elderly patient who had undergone single chamber pacemaker implant 20 years previously, and presented with miliary tuberculosis involving the heart and pacemaker pocket [4]. The second is a child with congenital heart disease who had an implant of an epicardial pacemaker [5]. Several months later the patient presented with decreased general health and moderate fever associated with a lump at the pacemaker site. Complete drainage and surgical ablation of the pacemaker demonstrated acid-fast bacilli at microscopy, and PCR confirmed *M. tuberculosis* infection. The third case also occurred at the site of epicardial pacing wires, which had been placed 11 months previously during coronary artery bypass surgery [6]. Drainage of the associated abscess confirmed M. tuberculosis infection. In another case report [7] ICD was implanted in an elderly patient and after 6 months the patient presented with pain and swelling around the ICD site. Pus was aspirated, microscopy and culture confirmed tuberculosis, the device was explanted and ATT started. Small number of cases of pacemaker infections associated with other mycobacterial species has also been reported including *Mycobacterium fortuitum*, *Mycobacterium abscessus*, *Mycobacterium chelonae* and *Mycobacterium avium* complex [8-13]. Reports of Mycobacterial infections are summarised in Table 2.

*Mycobacterium tuberculosis* infection of pacemaker pocket is rare and the diagnosis is often delayed. None of our patients presented with constitutional symptoms and all were initially thought to represent bacterial wound infection. Tuberculosis was only considered when all investigations for bacterial infections were negative. It is important, however, for clinicians to be aware of the possibility of reactivation of tuberculosis after implantation in patients with a higher prevalence of latent infection, such as endemic countries like India or the elderly with compromised immune status like diabetes mellitus, alcoholism, HIV infection and on immunosuppressive therapy. Mycobacterial infection can occur due to seeding of bacteria in the device pocket at the time of the procedure or due to haematogenous spread from foci from other site that subsequently remain clinically silent in the vicinity of the device for several months and later activate and cause florid infection. Diagnosis can be made by AFB staining, culture of pus, and PCR assay, and successfully treated with a combination of surgical debridement and antitubercular treatment for 9-15 months.

## Conclusion

We conclude that if a patient presents with delayed pacemaker infection and when Gram stain and routine cultures are negative then we should always think about the possibility of mycobacterial pacemaker infection.

## Figures and Tables

**Table 1:**
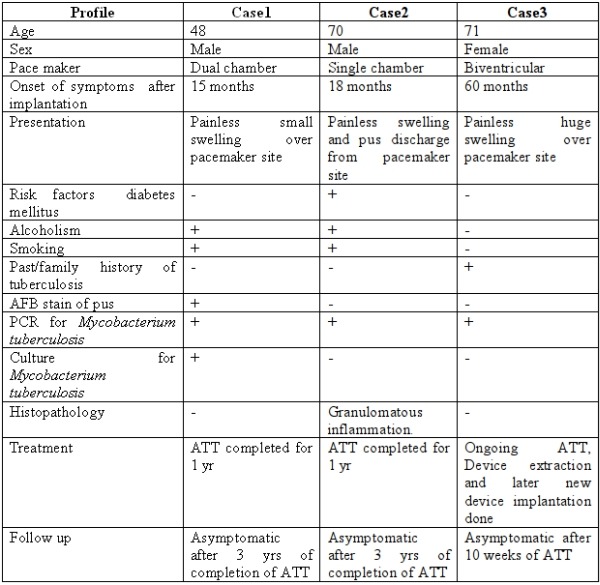
Characteristics of patient population.

**Table 2:**
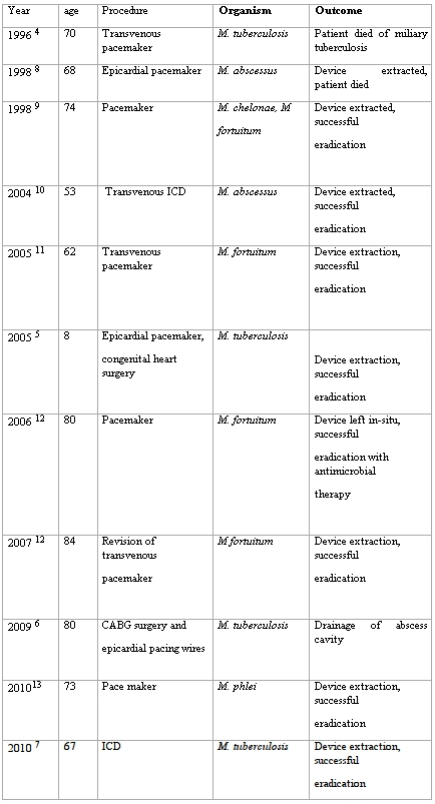
Mycobacterial infections of implantable devices as reported in literature.
